# Studies on immune regulation for allogeneic iPSC-based transplantation

**DOI:** 10.1186/s41232-022-00249-z

**Published:** 2022-12-30

**Authors:** Ken-ichiro Seino

**Affiliations:** Division of Immunobiology, Institute for Genetic Medicine, Hokkaido University, Sapporo, Japan

For allogeneic transplantation, immune regulation is critical, and the same is true for iPS cell-based cell and tissue transplantation. Since the first report of iPS cells in 2006, research has been conducted mainly on induction of differentiation into various functional cells for application to cell and tissue transplantation. Meanwhile, research on immune regulation seems to have been put on the back burner. However, nearly 15 years have passed since the invention of iPS cells, and light is finally being shed on issues related to immunity. In this context, research groups, mainly in Japan, have been studying this issue in recent years.

Kawamoto et al. of Kyoto University examined the possibility of graft injury by NK cells occurring in the HLA-homo state [1]. They showed a possible method of suppressing graft injury by NK cells using HLA-C.

Kaneko et al. of Kyoto University reported on gene editing technology to prevent the expression of HLA in iPS cells, describing not only the knockout of HLA but also the expression of HLA-E and CD47 to control NK cells [2].

Matsumoto et al. of Kyoto University summarized immune regulation in cardiac regenerative medicine using iPS cells [3]. They also discuss immunomodulation methods using activated Tregs and MSCs.

Finally, Seino et al. of Hokkaido University comprehensively reviewed immunoregulatory methods in iPS cell-based transplantation medicine [4]. In particular, they showed the mechanism of immune response when HLA-matched iPS cells are used and summarized the methods of controlling the immune response, from drug-based methods to cell-based methods, and genetic modification.

In organ transplantation, studies on immune tolerance have been conducted, in which grafts remain viable after discontinuation of immunosuppressive drugs. It has been shown that immune tolerance can be induced by means of molecular targeted therapy targeting molecules expressed on immune cells or by means of cell therapy using donor or recipient cells such as bone marrow. This has been achieved not only at the level of animal studies but also in the clinical setting, and such research directions should be important for iPS cell-based transplantation as well.

I hope that the progress of these studies will lead to safe transplantation medicine using allogeneic iPS cells and the development of this field.
